# A Memoir on Stricture of the Urethra

**Published:** 1850-07

**Authors:** 


*A Memoir on Stricture of the Urethra.* By J. P. Mettauer, M. D., LL. D., Professor of the Principles and Practice of Surgery in the Medical Department of Randolph Macon College, Virginia.

This monograph embraces an abstract of the personal experience of one, who has for many years occupied a prominent position in the medical profession, and who has devoted much time and careful attention to the study and treatment of stricture of the urethra. It is always gratifying, if not really instructive, to learn the results of the extended experience and numerous observations of those who have labored long and zealously in investigating the nature and ascertaining the most appropriate and successful modes of treating disease. The subject of which our author treats, is one that is often exceedingly perplexing to the practitioner and greatly annoying to the patient; not unfrequently requiring a great deal of patience and perseverance, as well as skill, on the part of the former and a determined co-operation on the part of the latter. That he has fully appreciated the many difficulties to be encountered in the proper management of this disease under the different phases in which it is seen in a large practice, and has availed himself of all the resources supplied by extensive reading, by careful observation, and much sound reflection, is evident from this account of the modes adopted and followed by him in his practice. After making some brief remarks respecting the varieties of stricture, he says, of their location in the urethra—" about two-thirds of my cases had their seats at or near the bulb. Fifty-one cases were attended with contractions at the bulb and anterior extremity of the prostate, and one hundred and eighteen in which the strictures occupied the posterior orifice of the fossa navicularis, the bulb and posterior extremity of the membranous portion of the urethra at the same time." In one hundred and eighty-two cases, treated successfully, sixteen were of the diaphragmatic variety, and " occurred at or near the orifices of the fossa navicularis or in the spongy portion of the urethra beyond the fossa.

The lesions constituting stricture and those consequent to it, as well as their symptoms, are briefly but clearly described. Much importance is very justly attached to lesions co-existent with, and consequent to stricture. The manner in which these are noticed, evinces a thorough and comprehensive knowledge of the subject of which the author treats. In exploring the urethra, to ascertain the seat of the stricture, the recumbent position, especially in difficult cases, is preferred, and the introduction of the sound with the convexity towards the symphisis invariably adopted, reversing it if it be found necessary to carry it beyond the bulb, without removing it from the urethra. In using the bougie, especially the waxen, to obtain the exact shape of the stricture, our author has found the erect posture generally preferable. Having gained the stricture, the bougie is allowed to remain twenty minutes or more before it is withdrawn. It seems, however, that he has, for some years past, abandoned for the most part the critical exploration of stricture "as a needless refinement in the diagnosis of the disease." He says:—"It certainly saves patients some bodily suffering, and not a little mortification from exposure of their genitals!" There are some cases, however, which he deems it necessary to explore thoroughly and with the utmost precision. The bridle variety is of this class.

In the constitutional treatment of stricture the use of nitro-muriatic acid, externally and internally, has been found a "most valuable means of regulating the bowels, after purging once or twice;" also a combination of Dover's powders and ox or swine's gall to obtain the same effect, in addition to sleep. Our space will not allow of a detailed account of the author's different modes of operating for the radical cure of stricture, or of the different instruments which he is in the habit of using, a minute description of which, accompanied with drawings, has been furnished in the pamphlet before us.

When more than one stricture exists he is in the habit of dividing them all before withdrawing the instrument, unless fainting or sickness of the patient renders it necessary. In some instances the incisions have been dilated by forcing a metallic bougie of a large size through the imperfectly divided stricture. This, however, is deemed a hazardous practice, especially in the hands of inexperienced operators. After the division of a stricture and the introduction of a tube the patient is placed in bed and required to remain there, generally, for several days—not less than two, unless the presence of the tube in the urethra should prove too irritating. The importance of using the bougie, occasionally, for some months, or even years, after the operation is strongly urged. Having noted some of the most important points in our author's valuable memoir on stricture of the urethra, sufficient, we trust, to give the reader a general idea of its character, and induce him to obtain it and peruse it for himself, we may be allowed to express the hope that his able pen may be spared many years yet to record for the benefit of his professional brethren the result of his individual experience.

